# Optimization of sonication time, edible coating concentration, and osmotic solution °Brix for the dehydration process of quince slices using response surface methodology

**DOI:** 10.1002/fsn3.3382

**Published:** 2023-04-21

**Authors:** Fakhreddin Salehi, Kimia Goharpour, Helia Razavi Kamran

**Affiliations:** ^1^ Department of Food Science and Technology Bu‐Ali Sina University Hamedan Iran

**Keywords:** color indices, guar gum, mass reduction, soluble solids gain, sucrose, surface area

## Abstract

The goal of this work was to examine the effects of sonication time, edible coating concentration (with guar gum), and °Brix (sucrose solution) on the osmotic dehydration (OD) parameters (mass reduction, water loss, soluble solids gain, and rehydration ratio) and the appearance properties (color indices and surface area) of quince slices using a response surface methodology (RSM) approach based on the central composite design (CCD), for the optimization of the process. The process parameters, sonication treatment time (5–10 min; 40 kHz and 150 W), edible coating concentration using guar gum (0.05%–0.15%, w/w), and osmotic concentration using sucrose solution (20%–50%, w/w), were investigated and optimized for OD of quince slices. After each OD process, the quince slices were dehydrated in an oven at 70°C for 240 min. Results demonstrated a good correlation between empirical data with the linear model. Using the optimization method, optimum input operating conditions were determined to be a sonication time of 5 min, guar gum concentration of 0.05%, and sucrose concentration of 37.19°Brix. At this optimum point, the OD process of quince slices reached the optimal mass reduction (17.74%), water loss (25.77%), soluble solids gain (8.03%), rehydration ratio (206.19%), lightness (77.6), redness (0.60), yellowness (34.84), total color change (Δ*E*) (8.92), and area changes (7.59%).

## Graphical abstract

The goal of this work was to examine the effects of sonication time, edible coating concentration (with guar gum), and °Brix (sucrose solution) on the osmotic dehydration parameters (mass reduction, water loss, soluble solids gain, and rehydration ratio) and the appearance properties (color indices and surface area) of quince slices using a response surface methodology approach based on the central composite design, for the optimization of the process.

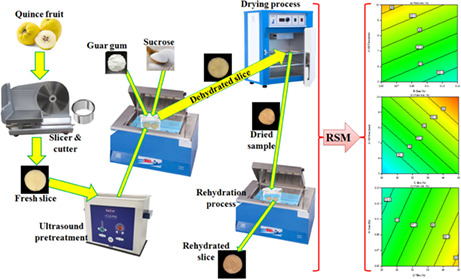

## 1 INTRODUCTION

Quince (*Cydonia oblonga*) from Rosaceae family is a tree cultivated as a medicinal and nutritional plant. This fruit is a rich source of vitamins A, B, and C, fibers, and tannin. Dehydration is one of the main preservation methods employed for the storage of quince fruit. Fresh and dehydrated quince fruit are used to make jam, marmalade, jelly, and quince pudding (Dehghannya et al., 2018; Salehi & Kashaninejad, 2018). Osmotic dehydration (OD) has been used as a pretreatment for many fruits and vegetables. During OD, water is removed from tissue by putting the fruit particles in a sugar solution. OD technique decreases the thermal damage to either color or taste, prevents enzymatic browning, and results in better protection of the nutritional component during dehydration process (Dehghannya et al., 2018; Salehi, 2023). Recently, some researchers used the OD technique for improving the quality properties of dehydrated quince fruit and found that this technique is a suitable method for the pretreatment and dehydration of quince samples (Dehghannya et al., 2018; Kutlu, 2021; Turkiewicz et al., 2020). Turkiewicz et al. (2020) used OD technique as a pretreatment before drying Japanese quince fruit using a convective and vacuum microwave dryer. Their results showed that the OD process significantly shortened the combined drying time compared with non‐OD samples.

Using pretreatment such as ultrasound has been shown to reduce OD time and increase water loss during OD (Salehi, 2023). Ultrasound pretreatment accelerates the mass transfer in dehydration and drying of fruit and vegetable slices mostly due to the breakdown of cells and the creation of microchannels (Salehi, 2020). A sonication frequency range of 20 kHz to 1 MHz is usually used in fruits and vegetables processing due to its physicochemical effect (Allahdad et al., 2019). The influence of pretreatment osmotic–ultrasonic dehydration on the rehydration kinetic of quince at three temperatures was investigated by Noshad et al. (2012). The results of this study showed that the osmotic–ultrasonic dehydration pretreatment caused samples with lower water absorption ability in comparison with the untreated samples because of cell permeabilization due to the process stress.

Coatings with edible gums have been widely analyzed, aiming to improve the quality and shelf‐life of fruit and vegetable products and decrease the soluble solids gain during OD. In addition, they used food slices for dehydration, a technique that can increase the nutritional and sensorial attributes of dehydrated products (Salehi & Satorabi, 2021). Guar gum is the powdered endosperm of the seeds of *Cyamopsis tetragonolobus*, which is a leguminous crop. It is a natural polysaccharide with non‐toxicity, safety, quick solubility in cold water, biodegradability, biocompatibility, lower prices, and easy availability characteristics. Due to its various functional characteristics, it is a promising biopolymer for the development of packaging or edible coating films (Thombare et al., 2016).

Response surface methodology (RSM), comprising sophisticated mathematical and statistical techniques, is used for the improvement and optimization of different processes (Roshanpour et al., 2023; Yang et al., 2023). In a study, Eren and Kaymak‐Ertekin (2007) proposed optimal conditions using a RSM test setup for OD of potato as 22°C for temperature, 54.5% for sucrose concentration, 14% for salt concentration, and 329 min for treatment time. At this optimal point they could achieve 52.9% mass reduction (MR), 59.1% water loss, 6.0% soluble solids gain, and 0.785 water activity. In another study, Bchir et al. (2020) proposed optimal conditions using a RSM test setup for ultrasound‐assisted OD of pomegranate seeds as 41°C for temperature, 60°Brix for sucrose concentration, and 240 min for treatment time. At this optimal point they could achieve 27.39% MR, 31.7% water loss, 4.25% soluble solids gain, and 7.39 × 10^−9^ m^2^/s effective moisture diffusivity (*D*_eff_).

This study aims to examine the effect of ultrasound pretreatment on the OD kinetics of coated quince slices searching for the optimal operating conditions (immersion time in the ultrasound bath, concentration of guar gum, and concentration of osmotic solution) that maximize MR, water loss, rehydration ratio, lightness (L*), and yellowness (b*), and minimize solid gain, redness (a*), total color change (Δ*E*), and area changes (Δ*A*) using RSM. In this work, after each OD process, the quince slices were dehydrated in an oven at 70°C for 240 min.

## 2 MATERIALS AND METHODS

### 2.1 Fresh materials

Fresh quince fruits (*C. oblonga* L.) were purchased from the market at Hamedan, Iran. The fresh and uniform‐sized quinces with no external damage were selected, and with the aid of an industrial slicer (food slicer, model AF‐23; Girmi) and a stainless steel ring mold cutter were cut into 5‐mm thick and 3.28 cm diameter slices. The initial quince slices moisture content was 81.42% w.b. (moisture determination was performed in a Shimaz oven, at 70°C for 4 h).

### 2.2 Ultrasound pretreatment

To apply the sonication treatments on the quince slices, a Backer vCLEAN1‐L6 ultrasonic bath (Iran) was employed with a frequency of 40 kHz and a power of 150 watts. The tank of the device was filled with 5 L of distilled water and, then, after the temperature of the water reached to 20°C, the quince slices were placed directly in the bath. Sonication pretreatments were applied to the quince slices in two replicates.

### 2.3 Edible coating and osmotic dehydration procedure

**FIGURE 1:**
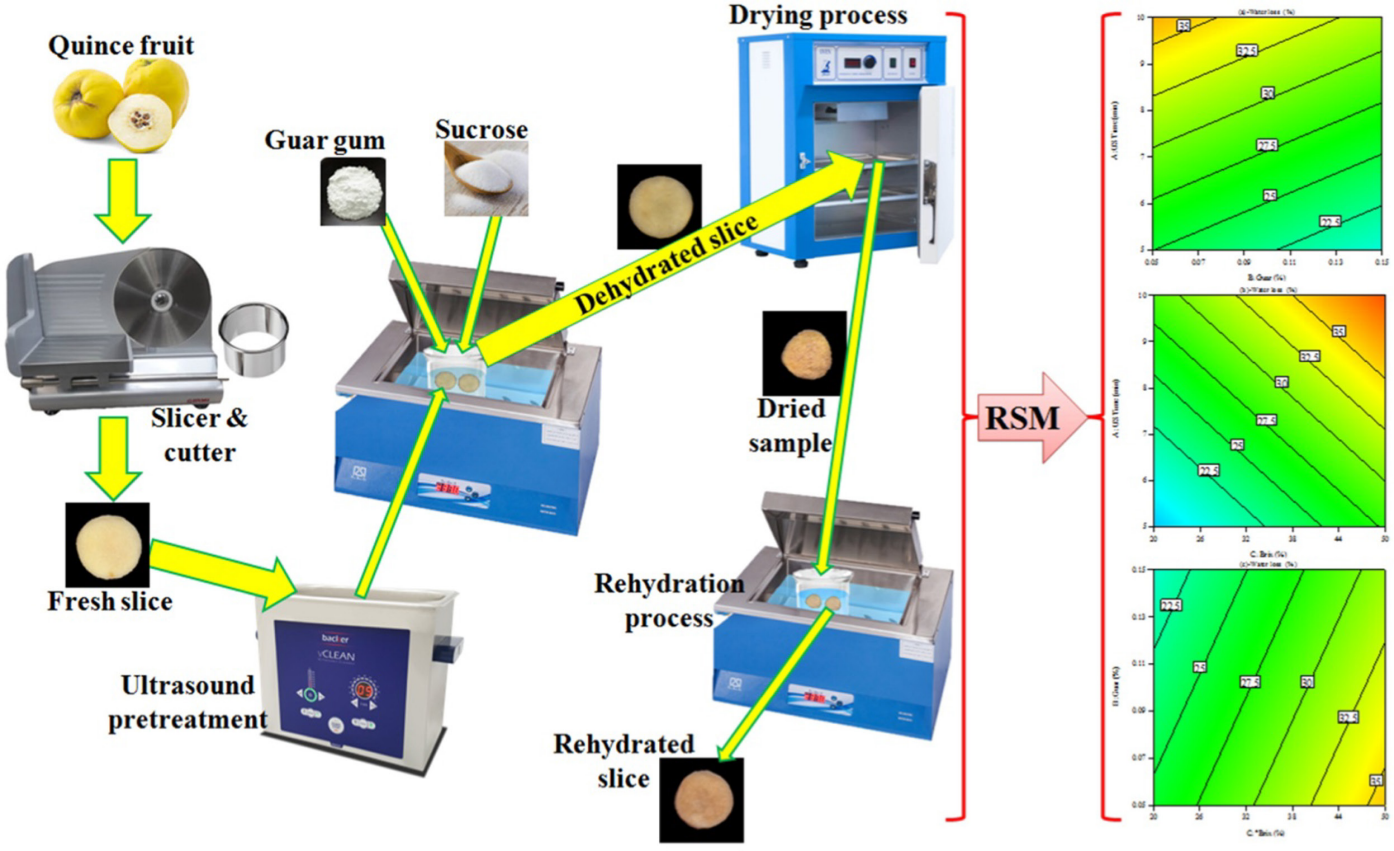
Schematic of the ultrasound pretreatment system and osmotic dehydration process of quince slices.

In this study, the treated samples with ultrasound were placed in a 200‐mL glass beaker containing sucrose and guar gum (in the determined concentrations), and then, beakers were placed in a water bath (R.J42; Pars Azma Co.) at 50°C for 60 min (Figure 1). After the termination of each experiment, quince slices were removed from the bath with forceps and wrapped immediately with a clean kitchen towel (microfiber towel) to remove excess moisture.

### 2.4 Air drying

The fresh and treated samples (before and after treatments) were dried (in single layer) at 70°C for 240 min using a laboratory scale dryer (Shimaz) equipped with temperature controllers and digital indicating controllers, an electrical heater, and a fan.

### 2.5 Rehydration ratio (RR) of quinces samples

Dried quince slices were weighed and immersed for 20 min in water (100 mL) at 50°C. The rehydration ratio was determined as the ratio of the final mass of samples (rehydrated quince slices) over the initially dried quince slices mass (after oven) (Salehi et al., 2022).

### 2.6 Mass reduction, water loss, and soluble solids gain

Before ultrasound treatment and after the osmotic, drying, and rehydration processes, the mass of the samples was measured using a digital balance (with an accuracy of ±0.01 g, model SL1000, Kia Laboratory, Iran). The dehydrated quince slices were analyzed for MR, water loss (WL), and soluble solids gain (SG) (Salehi et al., 2022).

### 2.7 Surface color and area measurement

Color measurement of food products has been used as an indirect measure of other quality attributes because it is simpler, faster, and correlates well with other physicochemical characteristics (Pathare et al., 2013). The color analysis of untreated (control) and treated quince slices were assessed from all the various pretreatment methods using the Image J software (V.1.42e), which gave L*, a*, and b* values (where L* represents lightness, a* represents redness, and b* represents yellowness). A scanner (HP Scanjet‐300) was used to photograph the quince slices. The fresh quince slices exhibited a yellow color, with L*, a*, and b* being 84.95, −1.02, and 33.14, respectively. The color variation was made with respect to the fresh sample. Equation 1 was used to compute the Δ*E* of the quince slice after OD (Salehi, 2019):

(1) $$\Delta E=\sqrt{{\left(\Delta L^{*}\right)}^{2}+{\left(\Delta a^{*}\right)}^{2}+{\left(\Delta b^{*}\right)}^{2}}$$

In addition, the surface area of the quince slice after OD was calculated using the threshold color plugin in the image analysis software (Image J, V.1.42e). The average surface area of fresh quince slices was 8.45 cm^2^. The area changes (Δ*A*) of the quince slice after the OD process was calculated using Equation 2:

(2) $$\Delta A=\frac{A_{0}-A_{t}}{A_{0}}\times 100$$

where Δ*A* is the area changes (%) and *A*_0_ and *A*_*t*_ (cm^2^) are the areas of fresh and dehydrated quince slices, respectively.

### 2.8 Experimental design and optimization by response surface methodology

In this study, we used Design Expert Version 13 software as a design and analysis tool to conduct experiments. Regression coefficients (RCs), the significance of the process variables, conformity of the experimental data to models, and optimal response variables can be obtained by using this software (Zhao et al., 2023). Sonication time (X1, 5–10 min), guar gum concentration (X2, 0.05%–0.15%, w/w), and sucrose concentration (X3, 20%–50%, w/w) were selected as independent variables through literature survey and preliminary experiments. The natural values and coded levels used in this study are presented in Table 1.

**TABLE 1:** Level of independent variables values used for osmotic dehydration process of quince slice.

Coded factor	Independent variables	Coded levels of variables		
		−1	0 (center point)	+1
A (X1)	Ultrasound time (min)	5	7.5	10
B (X2)	Guar gum concentration (%)	0.05	0.1	0.15
C (X3)	°Brix	20	35	50

The central composite design (CCD) is the most commonly used fractional factorial design used in the RSM model (Bhattacharya, 2021). In this article, we employed the CCD technique for designing the experimental collection. The design included 20 experimental points and it is adopted by adding 6 central points and 6 axial points to 8 full factorial designs (Table 2).

**TABLE 2:** Central composite design matrix.

Run	X1 (ultrasound time)	X2 (guar concentration)	X3 (°Brix)
1	7.5	0.02	35
2	10	0.05	50
3	7.5	0.18	35
4	5	0.15	20
5	7.5	0.1	35
6	5	0.05	50
7	7.5	0.1	35
8	7.5	0.1	9.77
9	10	0.05	20
10	7.5	0.1	35
11	10	0.15	20
12	10	0.15	50
13	7.5	0.1	35
14	7.5	0.1	60.23
15	7.5	0.1	35
16	3.30	0.1	35
17	5	0.05	20
18	11.70	0.1	35
19	7.5	0.1	35
20	5	0.15	50

This study aims to examine the influences of ultrasound pretreatment on the OD of coated quince slices to search for the optimum point (immersion time in the ultrasound bath, concentration of guar gum, and concentration of osmotic solution) that maximize MR, water loss, rehydration ratio, lightness, and yellowness, and minimize solid gain, redness, Δ*E*, and area changes using RSM (CCD). The experimental data were fitted to a second‐order polynomial model (Bchir et al., 2020; Yoo et al., 2023). The significant terms in the models were found by analysis of variance (ANOVA) for each dependent variable. The fitness of the model was probed via the comparison of the *F* test, lack of fit, RC, predicted RC, adjusted RC, and *p* value. Significance was judged by calculating the probability level that the *F* statistic estimated from the data is <5% (Roshanpour et al., 2023). The lack‐of‐fit values were checked corresponding to the variation of the data around the fitted model designed.

## 3 RESULTS AND DISCUSSION

### 3.1 Mass reduction

The linear model (suggested model) reveals the role of all independent variables in the dependent variables. The results of ANOVA revealed that the linear model was significant (.0001 < *p* < .04) for all dependent variables (Table 3). Also, the lack‐of‐fit values for all dependent variables are not significant (*p* > .05) relative to the pure error, showing good response to the linear model. Table 3 shows that the linear term of sonication time had the most significant (*p* < .05) effect on the MR of quince slices during the OD process. While the linear terms of guar gum concentration and °Brix have no significant (*p* > .05) effects on the MR of quince slices subjected to OD.

**TABLE 3:** Analysis of variance for the linear model for osmotic dehydration process of quince slices.

Source	Response 1: Mass reduction					Response 2: Water loss					Response 3: Solid gain				
	Sum of squares	df	Mean square	*F*	*p*	Sum of squares	df	Mean square	*F*	*p*	Sum of squares	df	Mean square	*F*	*p*
Model	405.03	3	135.01	6.33	.0049	862.86	3	287.62	8.06	.0017	120.53	3	40.18	7.41	.0025
A – Time	287.18	1	287.18	13.46	.0021	435.10	1	435.10	12.19	.0030	15.31	1	15.31	2.82	.1123
B – Guar gum	40.88	1	40.88	1.92	.1853	75.34	1	75.34	2.11	.1655	5.23	1	5.23	0.9640	.3408
C – °Brix	76.97	1	76.97	3.61	.0757	352.43	1	352.43	9.88	.0063	99.99	1	99.99	18.44	.0006
Residual	341.31	16	21.33			570.89	16	35.68			86.75	16	5.42		
Lack of fit	301.00	11	27.36	3.39	.0939	490.89	11	44.63	2.79	.1337	76.52	11	6.96	3.40	.0938
Pure error	40.30	5	8.06			79.99	5	16.00			10.23	5	2.05		
Cor total	746.34	19				1433.75	19				207.28	19			
	**Response 4: Rehydration ratio**					**Response 5: Lightness**					**Response 6: Redness**				
Model	9023.24	3	3007.75	3.49	.0404	166.27	3	55.42	16.56	<.0001	103.25	3	34.42	5.51	.0086
A – Time	3571.40	1	3571.40	4.14	.0587	12.01	1	12.01	3.59	.0764	25.62	1	25.62	4.10	.0599
B– Guar gum	1839.94	1	1839.94	2.14	.1633	85.82	1	85.82	25.64	.0001	51.64	1	51.64	8.27	.0110
C – °Brix	3611.91	1	3611.91	4.19	.0574	68.45	1	68.45	20.45	.0003	25.99	1	25.99	4.16	.0582
Residual	13,787.20	16	861.70			53.56	16	3.35			99.95	16	6.25		
Lack of fit	12,412.18	11	1128.38	4.10	.0655	37.80	11	3.44	1.09	.4952	87.69	11	7.97	3.25	.1018
Pure error	1375.02	5	275.00			15.76	5	3.15			12.26	5	2.45		
Cor total	22,810.44	19				219.84	19				203.20	19			
	**Response 7: Yellowness**					**Response 8: Total color change (Δ*E*)**					**Response 9: Area changes (Δ*A*)**				
Model	176.95	3	58.98	3.58	.0374	238.22	3	79.41	9.90	.0006	38.99	3	13.00	26.57	<.0001
A – Time	113.61	1	113.61	6.90	.0183	54.78	1	54.78	6.83	.0188	4.38	1	4.38	8.96	.0086
B – Guar gum	30.52	1	30.52	1.85	.1923	62.17	1	62.17	7.75	.0133	19.90	1	19.90	40.68	<.0001
C – °Brix	32.83	1	32.83	1.99	.1772	121.26	1	121.26	15.12	.0013	14.71	1	14.71	30.07	<.0001
Residual	263.55	16	16.47			128.31	16	8.02			7.83	16	0.4891		
Lack of fit	201.55	11	18.32	1.48	.3499	96.75	11	8.80	1.39	.3765	6.22	11	0.5654	1.76	.2766
Pure error	62.00	5	12.40			31.56	5	6.31			1.61	5	0.3212		
Cor total	440.50	19				366.53	19				46.81	19			

The coefficients of linear equation (actual factors) for the estimation of OD parameters of quince slices were reported in Table 4. The coefficient values of MR of quince slices subjected to OD show the maximum positive portion of sonication time (*β*_1_ = 1.834) followed by sugar concentration (*β*_3_ = 0.158). In addition, the appropriate model as fitted corresponds to Equation (3):

(3) $$\mathrm{MR}=4.412+1.834\;\text{Time}\;\left(\min\right)‐34.601\;\text{Guar}\;\left(\%\right)+0.158{}^{\circ}\text{Brix}$$

**TABLE 4:** Coefficients of linear equation (actual factors) for the estimation of osmotic dehydration parameters of quince slices.

	Mass reduction	Water loss	Solid gain	Rehydration ratio	Lightness	Redness	Yellowness	Total color change	Area changes
Constant	4.412	4.239	−0.171	267.243	76.432	−0.659	38.262	10.174	10.240
Time×	1.834	2.258	0.423	−6.469	−0.375	0.548	−1.154	0.801	0.227
Guar×	−34.601	−46.974	−12.373	232.143	−50.136	38.890	−29.896	42.673	−24.141
°Brix×	0.158	0.339	0.180	−1.084	0.149	−0.092	0.103	−0.199	−0.069

**FIGURE 2:**
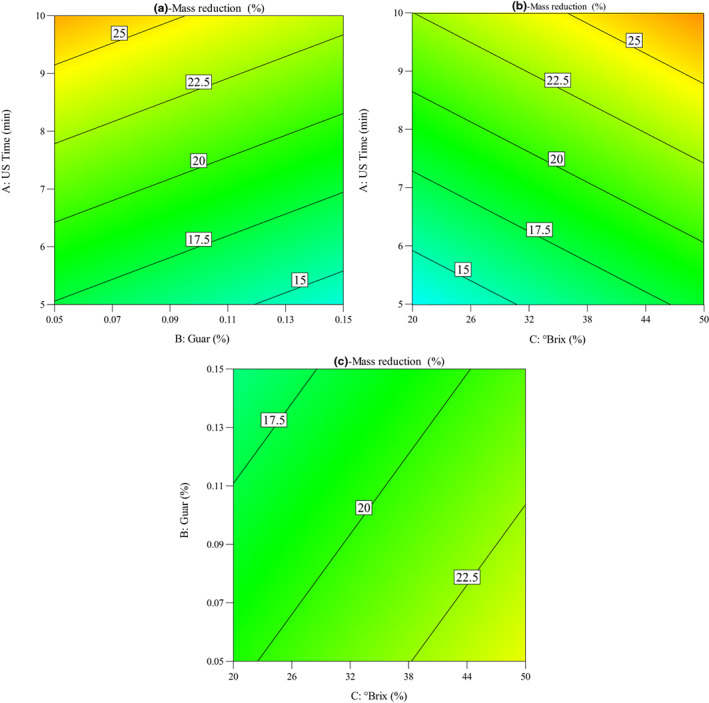
Contour plots for the impacts of operating conditions on the mass reduction of quince slices: (a) ultrasound time versus guar concentration, (b) ultrasound time versus °Brix, and (c) guar concentration versus °Brix.

Impacts of OD parameters (ultrasound time, guar concentration, and °Brix) on the changes in MR (%) of quince slices during the OD process were seen in Figure 2. The MR increased with the increase in sonication time. In addition, this figure shows the influence of guar gum concentration and sucrose solution concentration on the MR (%) of quince slices during OD. It was observed that MR decreased with the increase in guar gum concentration from 0.05% to 0.15%, while it increased with the increase in sucrose solution concentration from 20 to 50°Brix. Bchir et al. (2020) reported the same results showing that the ultrasound pretreatment and the osmotic solution °Brix (containing sucrose) are significant factors influencing the MR of pomegranate seeds.

### 3.2 Water loss

Edible coatings are thin layers of edible material added on the surface of fruits to improve the appearance, maintain the quality, decrease water loss, and extend shelf‐life (Odetayo et al., 2022). Results of various runs of OD of quince slices are shown in Table 3. An ANOVA was performed to characterize the significant impact of process variables on each dependent variable. The data from this table show that the sonication time has the major influences on the water loss of quince slices during the OD process. Also, the linear term of °Brix has a significant (*p* < .05) effect on the water loss of quince slices subjected to OD. Çağlayan and Barutçu Mazı (2018) reported similar results showing that the pretreatment time (for 40, 80, and 120 min) and the osmotic solution concentration (containing 40% and 60% sucrose) are significant factors affecting MR, water loss, and soluble solids gain of pumpkin slices. The linear term of guar gum concentration did not has a significant (*p* > .05) impact on the water loss of quince slices. The RCs (Table 4) show the maximum positive portion of sonication time (*β*_1_ = 2.258) followed by sugar concentration (*β*_3_ = 0.339) on the water loss of quince slices. These results reveal an enhancement in water loss with an increase in sonication time due to the greater water removal from samples. In addition, the appropriate model as fitted corresponds to Equation (4):

(4) $$\mathrm{WL}=4.239+2.258\;\text{Time}\;\left(\min\right)‐46.974\;\text{Guar}\;\left(\%\right)+0.339{}^{\circ}\text{Brix}$$

**FIGURE 3:**
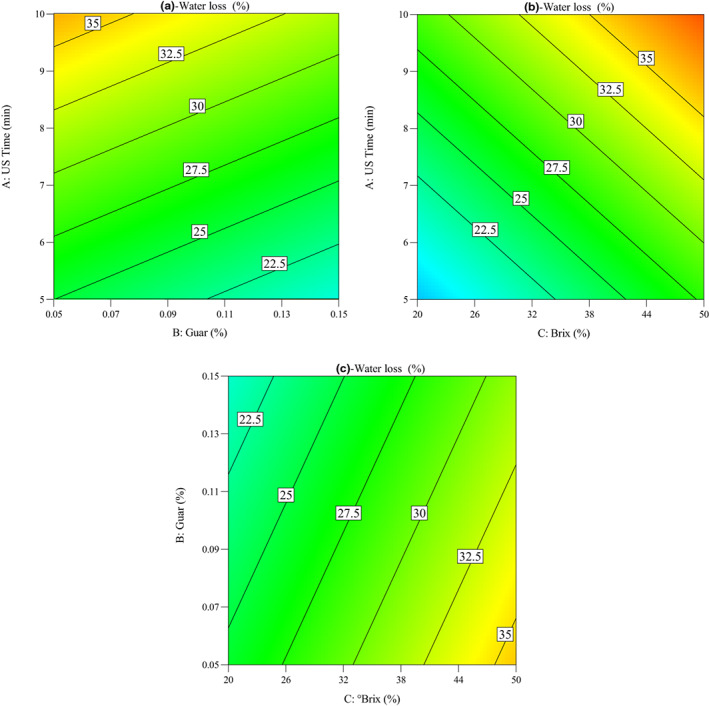
Contour plots for the impacts of operating conditions on the water loss of quince slices: (a) ultrasound time versus guar concentration, (b) ultrasound time versus °Brix, and (c) guar concentration versus °Brix.

Impacts of OD parameters on the changes in water loss (%) of quince slices during the OD process were seen in Figure 3. As shown in the figure, the ultrasound time, guar concentration, and °Brix plays important role in the water loss of quince slices. The sonication pretreatment increases the mass transfer of water from the quince slices into the osmotic solution by combining the effects of osmotic pressure and acoustic cavitation (Bchir et al., 2020). It was considered that water loss increased with the increase in sonication time from 5 to 10 min. Bozkir et al. (2019) confirmed that with increasing sonication time, the semipermeable membrane of the fruits weakened, thus allowing more water loss. The results of Barman and Badwaik (2017) demonstrated that with increase in ultrasonic treatment time during OD, the water loss and rehydration ratio of carambola (*Averrhoa carambola* L.) slices were increased. In addition, Figure 3 shows the influence of guar gum concentration and sucrose solution concentration on the water loss (%) of quince slices during OD. It was observed that water loss decreased with the increase guar gum concentration from 0.05% to 0.15%, while it increased with the increase in sucrose solution concentration from 20 to 50°Brix.

### 3.3 Soluble solids gain

The results of Table 3 demonstrate that the sugar concentration has the highest significant (*p* < .05) influence on the soluble solids gain of quince slices during the OD process. However, linear terms of sonication time and guar gum concentration did not have significant (*p* > .05) impacts on the soluble solids gain of quince slices. The RCs (Table 4) show the maximum positive portion of sonication time (*β*_1_ = 0.423) followed by sugar concentration (*β*_3_ = 0.180) on the soluble solids gain of quince slices. The result exposed an improvement in soluble solids gain with an increase in sonication time due to the greater mass transfer into the samples. In addition, the appropriate model as fitted corresponds to Equation (5):

(5) $$\mathrm{SG}=-0.171+0.423\;\text{Time}\;\left(\min\right)‐12.373\;\text{Guar}\;\left(\%\right)+0.180{}^{\circ}\text{Brix}$$

**FIGURE 4:**
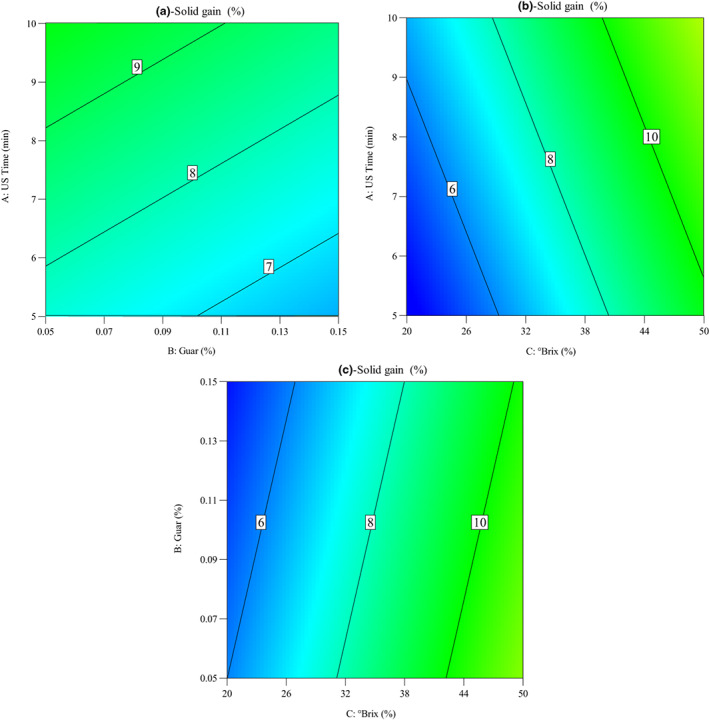
Contour plots for the impacts of operating conditions on the soluble solids gain of quince slices: (a) ultrasound time versus guar concentration, (b) ultrasound time versus °Brix, and (c) guar concentration versus °Brix.

The variation in the osmotic driving force among the osmotic solution containing sucrose and quince cells allowed the transfer of the sucrose solutes into the cells of quince slices. In addition to the osmotic pressure gradient, the sonication process could create microscopic channels in the cell membrane causing the transfer of the sucrose molecules into the quince cells (Corrêa et al., 2015; Salehi, 2023). As shown in Figure 4, the OD parameters including ultrasound time, guar concentration, and °Brix play important role in soluble solids gain of quince slices. It was considered that soluble solids gain increased with the increase in sonication time from 5 to 10 min. Similar results were also obtained by Kek et al. (2013), Alizadeh Shahrivar et al. (2017), Azarpazhooh et al. (2019), and Salehi et al. (2022), indicating that the ultrasound pretreatment should be considered due to its obviously improved soluble solids gain. Additionally, it was observed that soluble solids gain (%) of quince slices during OD decreased with the increase in guar gum concentration from 0.05% to 0.15%, while it increased with the increase in sucrose solution concentration from 20 to 50°Brix.

### 3.4 Rehydration ratio

Rehydration of food particulates is one of the qualitative parameters in the dehydrated product and aimed at the restoration of raw material properties when dehydrated material comes in contact with water. Rehydration is a complex phenomenon affected by numerous factors that typically include pretreatment methods, dehydration technique, food structure and composition, and medium viscosity (Marabi & Saguy, 2004; Noshad et al., 2012). The results of Table 3 demonstrate that the sonication time, guar gum concentration, and °Brix did not have significant (*p* > .05) effects on the rehydration ratio of quince slices during the OD process. The RCs demonstrate the maximum positive portion of guar gum concentration (*β*_2_ = 232.143) on the rehydration ratio of quince slices. In addition, the appropriate model as fitted corresponds to Equation (6):

(6) $$\mathrm{RR}=267.243-6.469\;\text{Time}\;\left(\min\right)+232.143\;\text{Guar}\;\left(\%\right)-1.084{}^{\circ}\text{Brix}$$

**FIGURE 5:**
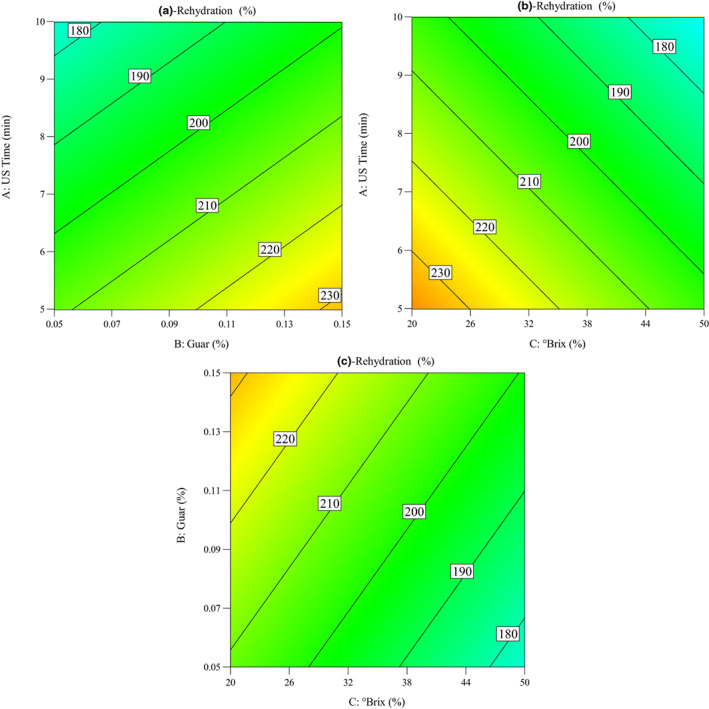
Contour plots for the impacts of operating conditions on the rehydration ratio of quince slices: (a) ultrasound time versus guar concentration, (b) ultrasound time versus °Brix, and (c) guar concentration versus °Brix.

Impacts of OD parameters on the changes in rehydration ratio (%) of quince slices during the OD process were seen in Figure 5. It was considered that the rehydration ratio decreased with the increase in sonication time from 5 to 10 min. In addition, this figure shows the influence of guar gum and sucrose solution concentration on the rehydration ratio (%) of quince slices during OD. It was observed that the rehydration ratio increased with the increase in guar gum concentration from 0.05% to 0.15%, while in was decreased with the increase in sucrose solution concentration from 20 to 50°Brix. The decrease in rehydration ratio of quince slices by increase in the sucrose solution concentration can be explained by a higher sucrose gain by samples. Sucrose may have entered into the microchannel, saturating the channel, decreased the pore size, and creating an extra resistance for water absorption during rehydration and therefore the rate of moisture absorption decreased significantly (Bakalis & Karathanos, 2005; Noshad et al., 2012).

### 3.5 Color indices

#### 3.5.1 Lightness

Color is a main quality parameter in the fresh and processed food products, and it influences consumer's choice and preferences (Pathare et al., 2013). The results of Table 3 demonstrate that the guar gum concentration has the highest significant influence (*p* < .05) on the lightness of quince slices during the OD process. Also, the linear term of °Brix has a significant (*p* < .05) effect on the lightness of quince slices subjected to OD. However, the linear term of sonication time did not had a significant (*p* > .05) impact on the lightness of quince slices. The RCs show the maximum negative portion of guar gum concentration (*β*_2_ = −50.136) followed by sonication time (*β*_1_ = −0.375) on the lightness of quince slices. In addition, the appropriate model as fitted corresponds to Equation (7):

(7) $$\text{Lightness}=76.432-0.375\;\text{Time}\;\left(\min\right)-50.136\;\text{Guar}\;\left(\%\right)+0.149{}^{\circ}\text{Brix}$$

**FIGURE 6:**
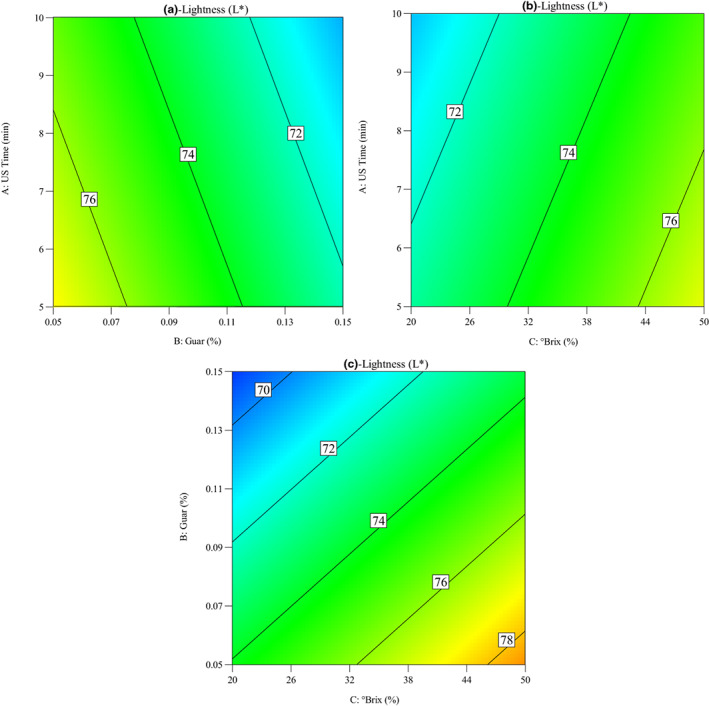
Contour plots for the impacts of osmotic dehydration parameters on the lightness index of quince slices: (a) ultrasound time versus guar concentration, (b) ultrasound time versus °Brix, and (c) guar concentration versus °Brix.

Impacts of OD parameters on the changes in the lightness of quince slices during the OD process were seen in Figure 6. As can be seen in Figure 6, the ultrasound time, guar concentration, and °Brix plays important role in the lightness of quince slices. It was considered that lightness decreased with the increase in sonication time from 5 to 10 min. These changes might be as a result of the combined effect of ultrasound waves and the osmotic solution that led to browning reaction and pigment degradation on the surface of the quince slices (Oladejo et al., 2017; Osae et al., 2019). Osae et al. (2019) stated that the lightness values of osmosonication treated ginger slices were significantly (*p* < .05) lower than that of the control (untreated) samples. In addition, Wiktor et al. (2016) reported lower lightness value of apple tissue treated with contact ultrasound compared to that of control (untreated sample).

In addition, Figure 6 shows the influence of guar gum concentration and sucrose solution concentration on the lightness of quince slices during OD. It observed that lightness decreased with the increase in guar gum concentration from 0.05% to 0.15%, while it was increased with the increase in sucrose solution concentration from 20 to 50°Brix.

#### 3.5.2 Redness

Table 3 shows that the linear term of guar gum concentration had the most significant (*p* < .05) effect on the redness of quince slices during the OD process. However, the linear terms of sonication time and °Brix did not have a significant (*p* > .05) effect on the redness of quince slices subjected to OD. The RCs show the maximum positive portion of guar gum concentration (*β*_2_ = 38.89) followed by sonication time (*β*_1_ = 0.548). In addition, the appropriate model as fitted corresponds to Equation (8):

(8) $$\text{Redness}=-0.659+0.548\;\text{Time}\;\left(\min\right)+38.89\;\text{Guar}\;\left(\%\right)-0.092{}^{\circ}\text{Brix}$$

**FIGURE 7:**
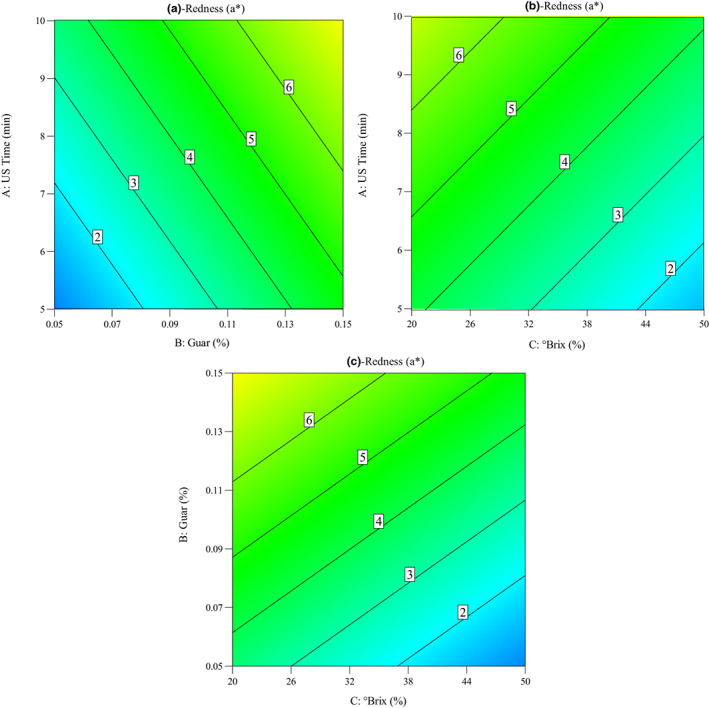
Contour plots for the impacts of osmotic dehydration parameters on the redness index of quince slices: (a) ultrasound time versus guar concentration, (b) ultrasound time versus °Brix, and (c) guar concentration versus °Brix.

The redness parameter of pretreated quince slices is shown in Figure 7. As can be seen in Figure 7, the redness index increased with the increase in sonication time from 5 to 10 min. In addition, this figure shows the influence of guar gum concentration and sucrose solution concentration on the redness of quince slices during OD. It was observed that redness increased with the increase in guar gum concentration from 0.05% to 0.15%, while it was decreased with the increase in sucrose solution concentration from 20 to 50°Brix. Deng and Zhao (2008) reported an increase in redness values of dehydrated apples cylinders (1.5 cm height × 1.5 cm diameter) pretreated in osmosonication process compared to the untreated samples.

#### 3.5.3 Yellowness

Table 3 shows that the linear term of sonication time had the most significant (*p* < .05) effect on the yellowness of quince slices during the OD process. However, linear terms of guar gum concentration and °Brix did not have a significant (*p* > .05) impact on the yellowness of quince slices subjected to OD. The RCs show the maximum negative portion of guar gum concentration (*β*_2_ = −29.896) followed by sonication time (*β*_1_ = −1.154) on the yellowness of quince slices. In addition, the appropriate model as fitted corresponds to Equation (9):

(9) $$\text{Yellowness}=38.262-1.154\;\text{Time}\;\left(\min\right)-29.896\;\text{Guar}\;\left(\%\right)+0.103{}^{\circ}\text{Brix}$$

**FIGURE 8:**
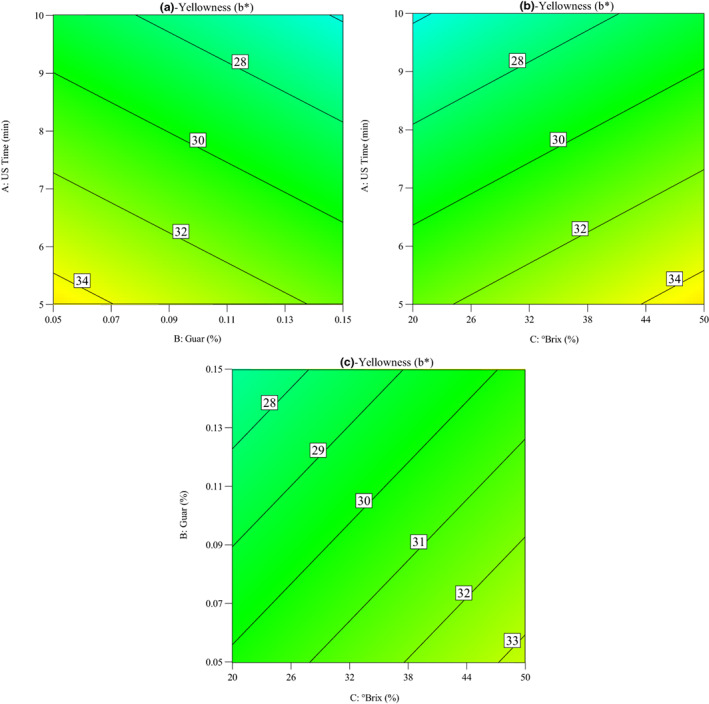
Contour plots for the impacts of osmotic dehydration parameters on the yellowness index of quince slices: (a) ultrasound time versus guar concentration, (b) ultrasound time versus °Brix, and (c) guar concentration versus °Brix.

Impacts of OD parameters on the changes in yellowness of quince slices during the OD process were seen in Figure 8. As shown in the figure, the ultrasound time, guar gum concentration, and °Brix plays important role in the yellowness of quince slices. It was considered that yellowness decreased with the increase in sonication time from 5 to 10 min. Also, it was observed that yellowness decreased with the increase in guar gum concentration, while it increased with the increase in sucrose concentration. Effect of ultrasound pretreatments on diffusion coefficients and color of osmodehydrated sweet potato was examined by Oladejo et al. (2017). Their results showed that the ultrasound treatment increased the lightness and decreased the redness of sweet potato, while osmosonication process enhanced the yellowness of sweet potato.

#### 3.5.4 Total color change

One of the most significant color parameter used to measure the color variation between fresh and processed food is the *ΔE* (Pathare et al., 2013). The results of Table 3 demonstrate that the sugar concentration has the highest significant (*p* < .05) influence on the Δ*E* of quince slices during the OD process. Also, the linear term of sonication time and guar gum concentration have significant effects (*p* < .05) on the Δ*E* of quince slices subjected to OD. The RCs demonstrate the maximum positive portion of guar gum concentration (*β*_2_ = 42.673) followed by sonication time (*β*_1_ = 0.801) on the Δ*E* of quince slices. In addition, the appropriate model as fitted corresponds to Equation (10):

(10) $$\Delta E=10.174+0.801\;\text{Time}\;\left(\min\right)+42.673\;\text{Guar}\;\left(\%\right)-0.199{}^{\circ}\text{Brix}$$

**FIGURE 9:**
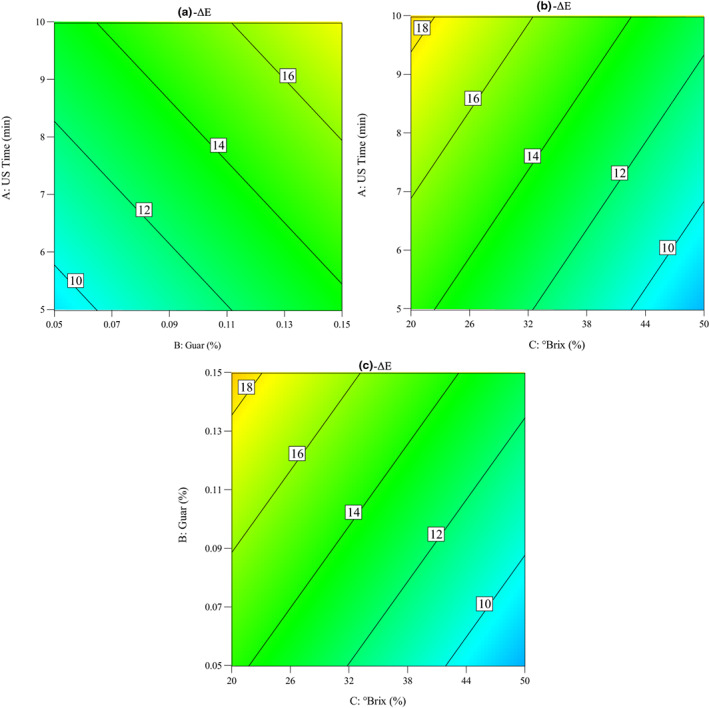
Contour plots for the impacts of osmotic dehydration parameters on the total color change index of quince slices: (a) ultrasound time versus guar concentration, (b) ultrasound time versus °Brix, and (c) guar concentration versus °Brix.

Impacts of the OD parameters on the changes in the *ΔE* of quince slices during the OD process were seen in Figure 9. It was considered that the *ΔE* increased with the increase in sonication time and guar gum concentration. Also, it was observed that the *ΔE* decreased with the increase in sucrose concentration. The effects of ultrasound, OD, and osmosonication pretreatments on color of dried ginger slices were investigated by Osae et al. (2019). The results of this study showed that the osmosonication pretreatment increased the *ΔE* of the dried ginger samples compared to ultrasound and OD. In addition, Oladejo et al. (2017) reported that the highest *ΔE* values were obtained in osmosonication pretreated sweet potato compared to the ultrasound and OD treated samples. Wiktor et al. (2016) stated that the *ΔE* values of sonicated apple tissue ranged from 1.2 to 9.8, whereas the changes were most quick in the case of contact ultrasound treatment.

### 3.6 Area changes

The values in Table 3 confirm that the sonication time, guar gum concentration, and °Brix have a significant effect (*p* < .05) on the area changes of quince slices during OD process. The RCs show the maximum negative portion of guar gum concentration (*β*_2_ = −24.141) followed by sugar concentration (*β*_3_ = −0.069) on the area changes (%) of quince slices. In addition, the appropriate model as fitted corresponds to Equation (11):

(11) $$\Delta A=10.24+0.227\;\text{Time}\;\left(\min\right)-24.141\;\text{Guar}\;\left(\%\right)-0.069{}^{\circ}\text{Brix}$$

**FIGURE 10:**
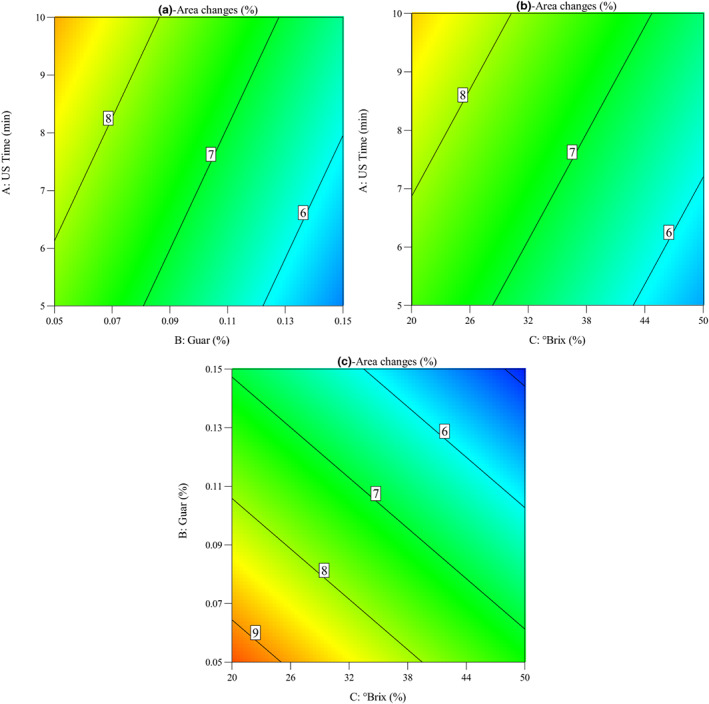
Contour plots for the impacts of osmotic dehydration parameters on the area changes (Δ*A*) of quince slices: (a) ultrasound time versus guar concentration, (b) ultrasound time versus °Brix, and (c) guar concentration versus °Brix.

Impacts of OD parameters on the area changes (%) of quince slices during OD process were seen in Figure 10. As shown in the figure, the ultrasound time, guar concentration, and °Brix plays important role in area changes of quince slices. It was considered that area changes increased with the increase in sonication time from 5 to 10 min. While it was observed that area changes (%) of quince slices during OD decreased with the increase in guar gum concentration and sucrose solution concentration from 0.05% to 0.15% and from 20 to 50°Brix, respectively.

### 3.7 The optimum condition

According to the design expert software optimization step, the desired target for each operational condition (sonication time, guar gum concentration, and °Brix) were chosen as “minimize and within the parameter range,” while the responses MR, water loss, rehydration ratio, lightness, and yellowness were defined as “maximize” and the responses soluble solids gain, redness, Δ*E*, and area changes were defined as “minimize.” It was observed that the optimum condition used for OD of quince slices was found to be sonication time = 5 min, guar gum concentration = 0.05%, and sucrose concentration = 37.19°Brix. In this condition, the OD of quince slices reached the optimal MR (17.74%), water loss (25.77%), soluble solids gain (8.03%), rehydration ratio (206.19%), lightness (77.6), redness (0.60), yellowness (34.84), Δ*E* (8.92), and area changes (7.59%).

## 4 CONCLUSIONS

Our results showed that the sonication time, guar gum concentration, and sucrose solution concentration effect on the MR, water loss, soluble solids gain, rehydration ratio, color indices (lightness, redness, yellowness, and Δ*E*), and area changes of quince slices subjected to OD. The optimum condition used for OD of quince slices were found to be sonication time = 5 min, guar gum concentration = 0.05%, and sucrose solution concentration = 37.19°Brix. The use of ultrasonic pretreatment increases the MR, water loss, and soluble solids gain and decreases the rehydration ratio of quince slices subjected to OD. The results showed that the rehydration ratio, redness, and Δ*E* parameters increased with enhancement in the guar gum concentration as an edible coating of quince slices. While the MR, water loss, soluble solids gain, lightness, yellowness, and area changes parameters decreased with enhancement in the guar gum concentration. The changes in osmotic solution Brix have significant effects (*p* < .05) on the water loss, soluble solids gain, lightness, Δ*E*, and area changes of quince slices.

## AUTHOR CONTRIBUTIONS

**Fakhreddin Salehi:** Conceptualization (equal); data curation (equal); formal analysis (equal); investigation (equal); software (equal); supervision (equal); validation (equal); writing – original draft (equal); writing – review and editing (equal). **Kimia Goharpour:** Data curation (equal); formal analysis (equal); investigation (equal); software (equal). **Helia Razavi Kamran:** Data curation (equal); formal analysis (equal); investigation (equal); software (equal).

## FUNDING INFORMATION

None.

## CONFLICT OF INTEREST STATEMENT

None.

## ETHICS STATEMENT

This study does not involve any human or animal testing.

## Data Availability

All data generated or analyzed during this study are included in this published article.
